# Utilization of e-mental-health and online self-management interventions of patients with mental disorders—A cross-sectional analysis

**DOI:** 10.1371/journal.pone.0231373

**Published:** 2020-04-20

**Authors:** Carolin Webelhorst, Lene Jepsen, Christine Rummel-Kluge

**Affiliations:** 1 Department of Psychosomatic and Psychotherapy, University of Leipzig, Leipzig, Germany; 2 Department of Psychiatry and Psychotherapy, University of Leipzig, Leipzig, Germany; University of Taipei, TAIWAN

## Abstract

**Background:**

Web-based treatments and online self-management interventions extend the range of therapeutic supply. Since the number of online self-management interventions is steadily increasing, we aimed to examine, how web-based services currently influence mental health care, asking about previous internet use and future interests of patients with mental disorders.

**Methods:**

We consulted patients (*n* = 400) from all services of the Department of Psychiatry and Psychotherapy of the University of Leipzig using a 28-item questionnaire. Overall, 301 questionnaires could be used for analysis. The data were analysed by means of descriptive statistics and group comparisons.

**Results:**

The majority of patients (98.3%) were using the internet. Data revealed younger patients were searching for information on diseases (*p* < .001; M = 35.7 ±13.2), psychiatrists (*p* < .001; M = 34.6 ±11.6) and exchange with other patients (*p* < .001; M = 32 ±10.6) more often than older patients. We also found the internet was consulted more often regarding the search for information *(p* = .011; M = 58.3 ±10.9) and psychiatrists (*p* < .001; M = 35.7 ±13.2) the lower the patients’ level of functioning was. While only a small proportion (10.1%) of the sample had used online self-management interventions before, there is a far greater number (46.1%) who stated an interest to use online self-management interventions in the future. This interest was greater in patients who were younger *(p* < .001; M = 33.8 ±13.2) had a higher education level (*p* = .003; university degree = 59.2%, high school degree = 52.3%; mandatory school degree: 34.8%).

**Conclusions:**

While only a small percentage of patients uses online self-management interventions, there is a far greater interest to include them into the treatment. Further research has to investigate how the integration of web-based services into the whole treatment process can be optimized. In addition, standardized diagnostic methods have to be found to evaluate the needs and experiences of patients.

## Introduction

The internet influences almost every part of our life. Both professional and personal activities are connected to the digital world–some improving, some complicating our lives [1]. In 2018, 90% of the German population were online [2]. This figure has been steadily increasing [3].

The health care system is one of the fields affected by the growth of digital technologies [4]. Web-based treatments and online self-management interventions extend the range of treatments, especially for those patients who are waiting for a conventional therapy or for those who live in areas with less medical supply [5]. The internet is not only a source of information, but it also provides a growing number of interventions to help patients cope with their liabilities [6]. Several studies show the effectiveness of self-guided, internet-based cognitive behavioural therapy in the treatment of mental disorders, especially for patients suffering from anxiety disorders and/or depression, while clinician-guided online-interventions show even better effect sizes on measures of depressive symptoms [7–9]. Such interventions range from preventive and informational to self-help, treatment as well as aftercare. Yet the rapidly growing number of online self-management providers available and the lack of standardized clinical validation regarding their efficiency and safety can be challenging and intimidating to patients [10–12]_._ Because of these facts, several countries in Europe are engaging in digital development and E-Health programs such as eMEN—a project funded by the European Regional Development Fund–which focusses on the prevention of mental health problems using self-management interventions [13]. Another project, ImpleMentAll, is aiming to implement E-Health interventions into the health care systems of different countries [14]. To regulate data protection and quality standards, Germany introduced an E-Health law in 2015 and revised it 2018 [15,16]. This legal framework also aims to reduce bureaucracy in the health care system, while the United Kingdom, the Netherlands or the Scandinavian nations are already working with elaborate E-Health services that are more integrated into their health care systems [17]. Yet, none of these efforts have reached their full potential [18]. Beside these infrastructural obstacles the success of web-based therapy also depends on the willingness of the patient and the attitude of the professionals, adding further complexity to the implementation of e-mental-health services [19,20].

In order to examine the interest for digital therapeutic services with the presented study, we aimed to determine the impact of internet use in patients with mental disorders in a sample of German patients, since the digital world is rapidly growing [21]. We also proposed to investigate the following characteristics, to determine how patients with mental disorders are currently using web-based therapeutic services:

1. Potential differences in internet use, considering demographic aspects among the patients
2. General internet use of patients with mental health disorders, with a particular focus on the possession of mobile devices (smartphone, tablet)
3. Patients’ experiences with online self-management interventions and interest in coping with their impairment
4. Potential differences in the use of online self-management interventions between varying diagnoses and the level of functioning

## Methods

### Study population

The study was conducted at the Department of Psychiatry and Psychotherapy of the University of Leipzig between January 2018 and January 2019. Patients consulted in this research at the time were being treated either as inpatients, outpatients, or at day hospital services. Inclusion criteria were: age ≥18 years, ability to agree to the inform consent, and ability to complete the questionnaire on their own. Exclusion criteria included illiteracy, insufficient German language skills, and a cognitive impairment (i.e. dementia) or an acute suicidal crisis. The study was approved by the Ethical Committee of the Medical Faculty of Leipzig University by November 3^rd^, 2017 and was registered at the German Clinical Trials Register under number DRKS00013332.

### Material

The questionnaire was originally developed for a study of the German public broadcasting cooperations (ARD/ZDF) which has been conducted annually since 1997 to investigate the internet behaviour of the general population in Germany with a representative sample [22]. A modified version was used by Kalckreuth et al. (2014) and Trefflich et al. (2014) with a similar clinical sample [23,24]. It was adjusted for the purpose of this study by adding further queries on the possession of mobile devices and online self-management services [25]. We decided to use this questionnaire to obtain descriptive values of a similar sample. A clinical diagnosis, therapeutic and medication data were assessed for all patients participating in the study by the attending professional. Additionally, the quantitative rating of the level of functioning was obtained by the attending physician using the Global Assessment of Functioning Scale (GAF) [26]. The total number of self-rated items assessed was 28. The first section included five socio-demographic questions, including gender, age, marital status, education level, and occupation using four multiple-choice and one open-ended question. The second section, including seven questions, assessed the general internet use with additional questions concerning possession of mobile devices to include the availability of mobile web-based therapeutic interventions. This section contained yes-or-no questions, as well as multiple-choice questions and open-ended questions. A third section contained 16 questions on mental health-related internet use, as well as the use of and demand for online self-management interventions, and an evaluation of those interventions. The third section also consisted of yes-or-no, multiple-choice and open-ended questions. All participants also took part in a simultaneous research on sexuality and sexual functions of patients with mental disorders. These results will be published elsewhere. The questionnaire is accessible in the Supporting Information section (S1 and S2 Files).

### Procedure

Inpatients and day hospital patients were approached after their ward rounds by independent study staff. After introducing them to the subject of the research and the questionnaire, they were included in the study if they met the inclusion criteria. Likewise, outpatient participants were orally addressed in the waiting room of the respective department by independent study staff. They were orally introduced to the study as well as the questionnaire and were included if they met the inclusion criteria. All participants were informed about the treatment and protection of their data and were given an information sheet about the study as well as a consent form regarding data protection. After an appropriate time to consider their agreement to participate, patients were given a questionnaire and had thirty minutes to complete it. The study staff has not been involved into the treatment of participating patients at any time to protect the privacy of the patients and the integrity of the treatment and prevent coercion.

### Data analysis

The data were treated confidentially and pseudonymized before evaluation. For all statistical analysis the statistical software IBM SPSS24 for Windows was used. Demographic data and main outcomes are reported using descriptive statistics. Answers to open-ended questions were discussed by the authors, sorted and classified into corresponding categories. Disagreement on the categorization between the authors were solved by majority decision. To analyse differences based on patients’ diagnoses, diagnoses were grouped according to the categories of ICD-10 [27]. To compare two independent samples (e.g. patient who possessed a smartphone vs. patients who did not possess a smartphone) regarding the variables age, duration of internet use, and the level of functioning (GAF), independent T-Tests were used. For all other variables (e.g. internet had helped patients to cope or did not help patient to cope with mental illness and gender, education level and diagnosis) χ^2^-tests were applied. To test differences in more than two samples (e.g. internet helped patients to cope, patients were not sure if the internet was helpful for coping or the internet did not help patients to cope with their mental illness) regarding the variables age and level of functioning (GAF) one-way Analysis of Variances (ANOVA) were performed. To assess the correlation between age and level of functioning, as well as duration of internet usage and age, the Pearson correlation coefficient was used. The significance level was set to α = .05. For multiple testing, the Bonferroni correction has been applied.

## Results

### Participation rate

In total, 400 patients were asked to participate in the study and inclusion Criteria were checked. Overall, n = 313 (78.3%) gave their consent to participate in the study by signing the consent form and were given the questionnaire in a further step. All n = 313 returned the questionnaires to the study staff. Of all submitted questionnaires, n = 12 participants had to be excluded from the study, since they returned empty questionnaires (n = 3) or were not capable of completing the questionnaire without help (n = 9). Thus, n = 301 (75.3%) of the questionnaires could be used for analysis. Due to left out answers, the number of participants might vary between questions. In these cases, they are declared in brackets.

### Sample characteristics

The sample consisted of n = 301 patients, of which 45.8% (n = 138) were inpatients, 26.6% (n = 80) outpatients, and 27.6% (n = 83) in day hospital treatment. The level of functioning was assessed by using the GAF scale, with a mean GAF of 57.4 (*SD* 11.7), which corresponds to the following category: “moderate symptoms and/or moderate difficulty in social, work or school functioning”.

Other demographic characteristics are shown in Table 1.

**Table 1:** Demographic and clinical characteristics of the sample.

Demographic characteristics	Mean (SD)	n (%)
	[Min, Max]	
**Gender (*n* = 301)**		
Female		167 (55.5)
Male		134 (44.5)
**Age (yr) (*n* = 301)**	37.3 (13.6)	
	[18,82]	
**Marital status (*n* = 300)**		
Unwed		170 (56.7)
Married/living with		89 (29.7)
Partner		
Divorced/separated		36 (12)
Widowed		5 (1.7)
**Education level (*n* = 297)**		
Without degree		6 (2)
Mandatory school		124 (41.8)
High school degree		93 (31.3)
University degree		74 (24.9)
**Occupation (*n* = 300)**		
Apprentice		13 (4.3)
Student		37 (12.3)
Unemployed		66 (22)
Employee/ official		117 (39)
Self-employed		3 (1)
Housewife/-husband		5 (1.7)
Retiree		41 (13.7)
Others (i.e. interns, volunteers)		18 (6)
**Clinical characteristics**	**Mean (*SD*)**	***n* (%)**
	**[Min, Max]**	
**GAF (Global Assessment of Functioning)**	57.4 (11.7)	
**(*n* = 301)**	[20,85]	
**Diagnoses according to**		
**ICD-10 (*n* = 301)**		
**F10-F19** (disorders due to psychoactive		11 (3.7)
substance use)		
**F20-F29** (schizophrenia, schizotypal, and		26 (8.7)
delusional disorders)		
**F30-F39** (affective disorders)		158 (52.5)
**F40-F49** (neurotic, stress-related, and		64 (21.3)
somatoform disorders		
**F50-F59** (behavioural disorders with physical		4 (1.3)
disorders)		
**F60-F69** (disorders of adult personality, and		23 (7.7)
behaviour)		
**F90-F98** (behavioural and emotional disorders		15 (5)
beginning in childhood and adolescence)		

yr = years; SD = standard deviation; n = sample size

### General internet use and mobile access

The mean duration of time spent on the internet was 20.6 hrs (*SD* 17.2 hrs; Min: 0.5 hrs; Max: 100 hrs) per week. The majority of participants (98.3%; *n* = 296) stated that they had used the internet before with a noticeable tendency to access the web from mobile devices such as smartphone (90.3%; *n* = 269) or tablet (36.6%; *n* = 109). Participants who possessed a smartphone were significantly younger than those who reported not to possess a smartphone (*t*(31) = -3.85, *p* = .001). Also participants owning a smartphone were statistically significantly less impaired as assessed by the GAF than patients who did not own a smartphone (*t*(31) = 2.2, *p* = .032). Patients who were possessing or not possessing a smartphone did not differ statistically significantly with regard to gender (χ^2^ (1, *N =* 298) = 0.72, *p* = .397), education level (χ^2^ (3, *N =* 294) = 4.22, *p* = .239), and diagnosis (χ^2^ (6, *N =* 298) = 9.39, *p* = .153). Further, we found older patients tended to use the internet for shorter durations than younger patients, to be shown in a negative significant correlation between duration of internet usage and age (*r(299)* = -.36, *p* < .001).

### Health and mental health-related internet use

**Fig 1:**
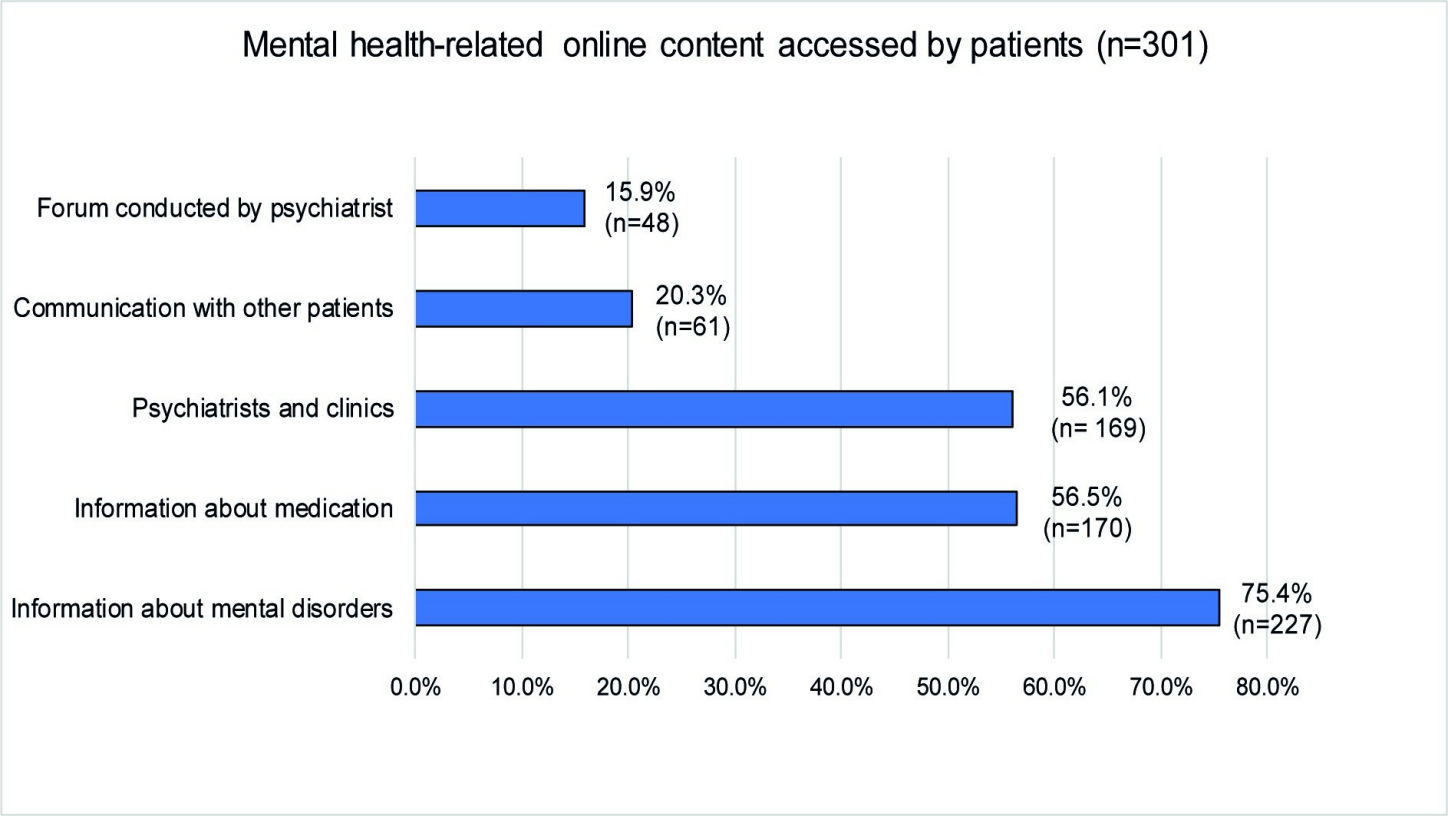
Web content used by the patients.

**Fig 2:**
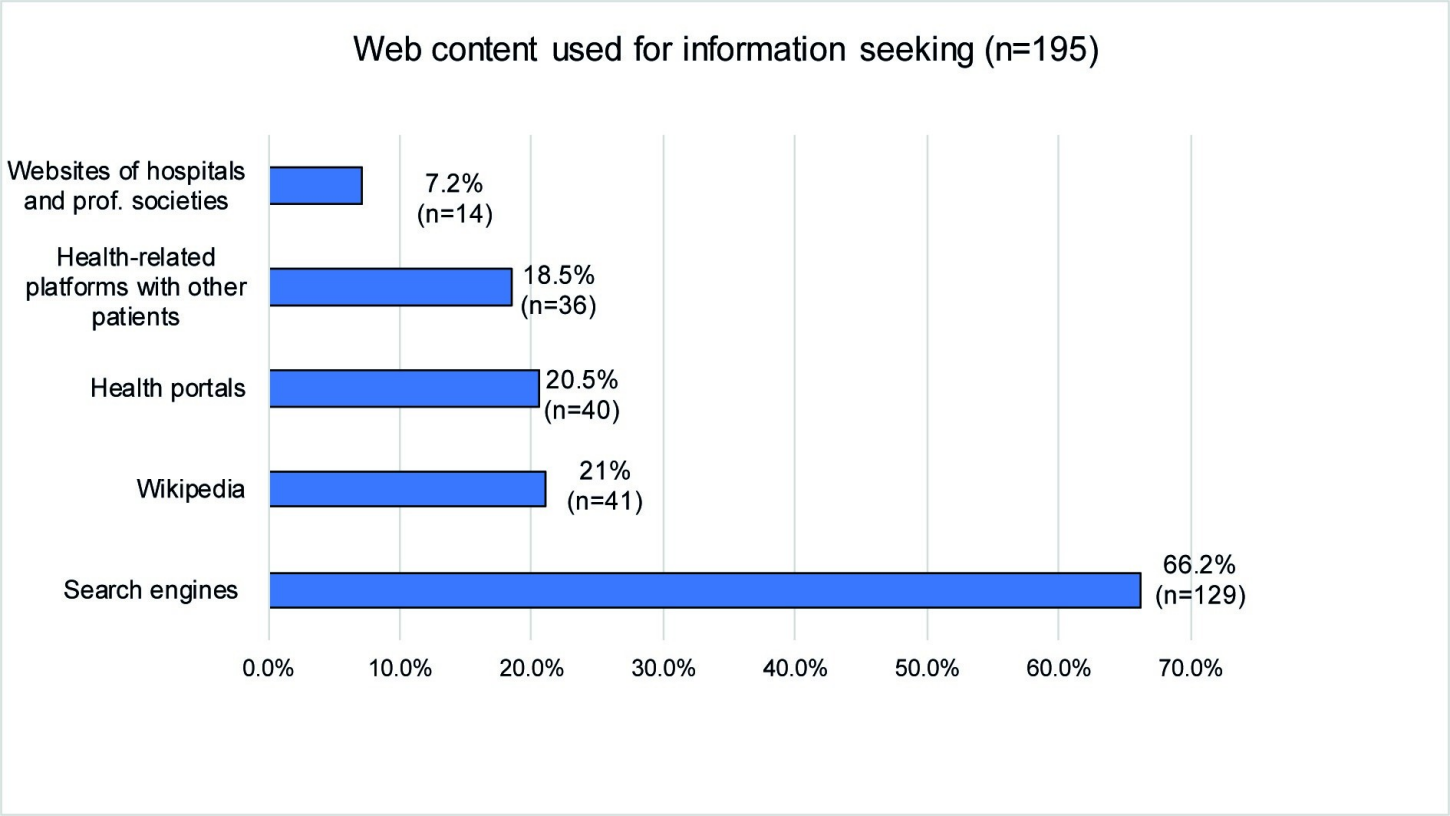
Mental health-related online use.

The internet was used by 89.3% (*n* = 266/298) of the sample to search for general health-related information (e.g. vaccination, symptoms) and by 88.8% (*n* = 262/295) for researching mental health-related issues (symptoms, therapy options). Those patients, who used the internet to inform themselves about mental disorders, utilized web content as shown in Fig 1. Mental health-related internet use is shown in Fig 2.

Patients who were searching for information on mental disorders (*t*(299) = 2.566, *p* = .011) and professional help by psychiatrists (*t*(254) = 3.87, *p* < .001) online had a statistically significantly lower level of functioning (GAF) than patients who were not using the internet in this regard. Patients who were searching for information on mental disorders (*t*(299) = -3.622, *p* < .001), psychiatrists (*t*(236) = -3.827, *p* = < .001) or exchange with other patients (*t*(119) = -4.073, *p* < .001) were significantly younger than patients who did not consult the internet on mental health-related topics. Patients who were searching for information on mental disorders (χ^2^ (6, *N =* 301) = 5.99, *p* = .424), medication (χ^2^ (6, *N =* 301) = 5.84, *p* = .442), psychiatrists (χ^2^ (6, *N =* 301) = 9.43, *p* = .151), or for exchange with other patients (χ^2^ (6, *N =* 301) = 12.11, *p* = .06), and those who were not searching for these contents, did not differ significantly with regard to different diagnoses.

### Coping with mental illness

A majority of participants (84.5%, *n* = 224/265) reported that the information found online were generally helpful in understanding their respective diagnosis, stating the following reasons: improved understanding of one’s mental illness (59.6%; *n* = 31/52), followed by exchange of experience (25%; *n* = 13/52), and insight into the illness (13.5%; *n* = 7/52).

**Fig 3:**
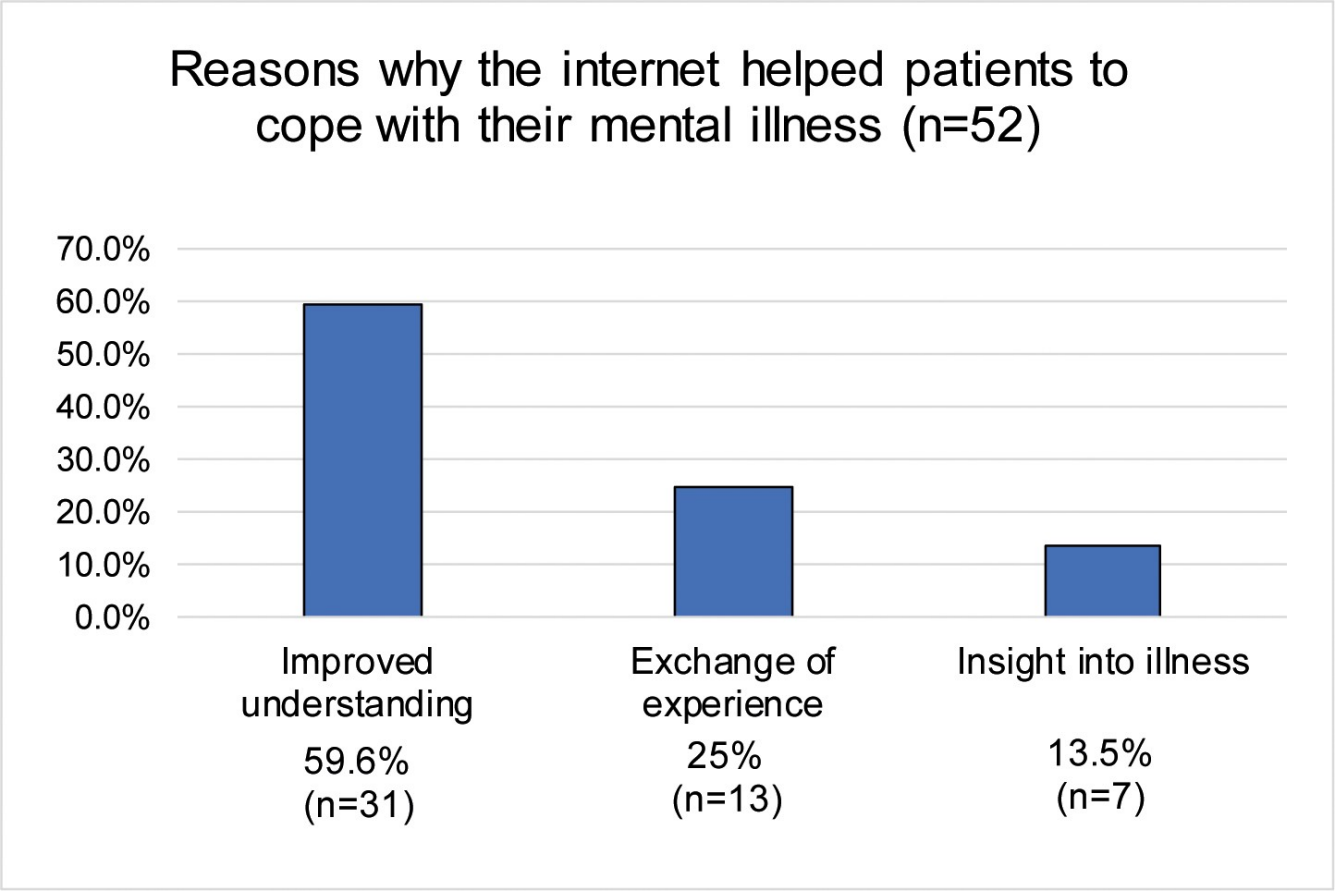
The internet's role in coping with mental illness.

**Fig 4:**
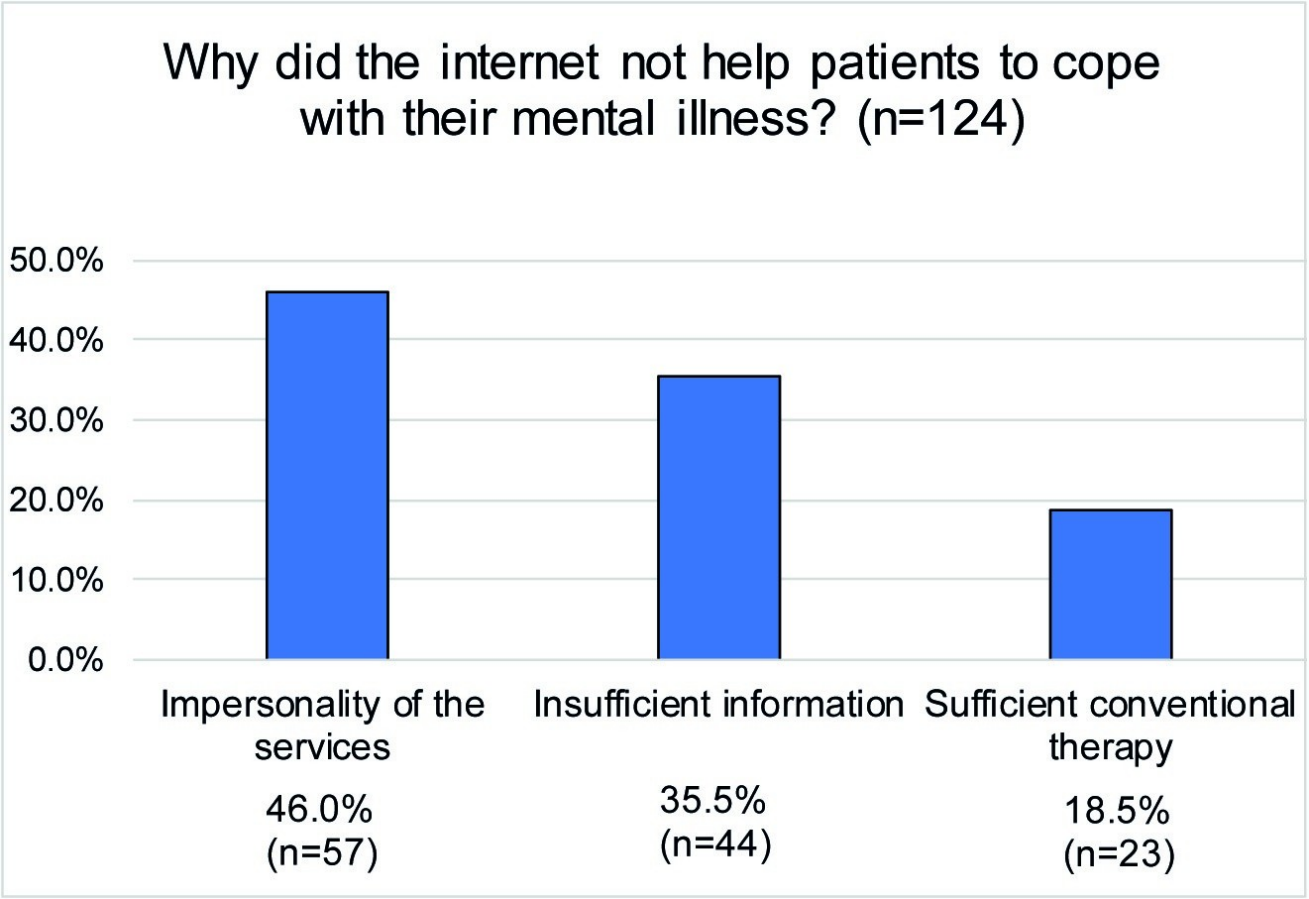
Concerns about mental health-related internet use.

Only 18.9% (*n* = 52/275) stated that the use of the internet helped them to cope with their mental illness. More than half of the sample (52.7%; *n* = 145/275) specified that the information found online did not help them to cope with their mental illness. Reasons why the internet helped or did not help the patients are shown in Figs 3 and 4. In addition, 28.4% (*n* = 78/275) of the sample were undecided about whether the internet helped them to cope with their mental illness or not. Patients who reported that the internet had helped them to cope with their mental illness did not differ from those who reported that the internet was not helpful to cope or from those patients who were undecided with regard to age (*F*(2,271) = .713, *p* = .491), gender (χ^2^ (2, *N =* 274) = 1.08, *p* = .582), education level (χ^2^ (6, *N =* 271) = 8.75, *p* = .188), level of functioning (*F*(2,271) = 2.249, p = .107) and diagnosis (χ^2^ (12, *N =* 274) = 10.32, *p* = .588).

### Online self-management interventions

Almost half of the sample (46.1%; *n* = 129/280) showed an interest to use online self-management interventions to cope with their mental disorders. Participants who reported an interest in online self-management interventions were statistically younger (*t*(278) = 3.519, *p* = < .001) and differed in their educational level (χ^2^ (3, *N =* 277) = 14.04, *p* = .003) compared to patients who were not interested in such interventions. These differences are evident between all educational levels and show the greatest variance between patients with a university degree and mandatory school degree: While more than half of the patients with a university degree (59.2%) had an interest for the use of online self-management interventions, only 34.8% of the patients with a mandatory school degree stated such an interest. Additionally, the interest in online self-management interventions was greater, in case online services already used by the patients had been experienced as helpful (χ^2^ (1, *N =* 253) = 4.54, *p* = .033) than in patients who had not experienced the internet as helpful. The data revealed no significant differences regarding the interest in online self-management interventions for different diagnoses (χ^2^ (6, *N =* 280) = 12.28, *p* = .056). The comparison of the interest in online self-management interventions among patients with different diagnoses showed the following outcome: The interest in online self-management interventions was smallest among patients suffering from affective disorders (37.2%; *n* = 55/148) compared to other groups of diagnoses: e.g. schizophrenia (52.2%; *n* = 12/33) and patients suffering from neurotic, stress-related and somatoform disorders (51.7%, *n* = 31/60). Patients with or without an interest in online self-management interventions did neither differ in gender (χ^2^ (1, *N =* 280) = 1.04, *p* = .309) nor in the level of functioning (*t*(278) = -1.036), *p* = .301).

**Fig 5:**
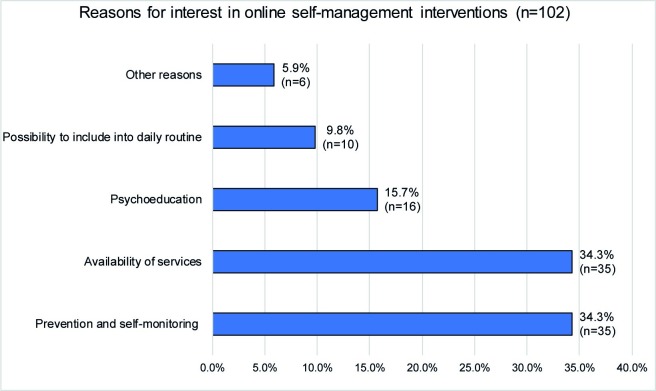
Reasons for online self-management interventions.

Only 10.1% (*n* = 30) had already used self-management applications (e.g. moodgym, iFightDepression, deprexis24) [28]. Participants who had used online self-management interventions showed a significant difference in gender: women seemed more likely to use online self-management interventions (χ^2^ (1, *N =* 298) = 10.32, *p* = .001). Patients who had used online self-management interventions and those who did not use such services, did not differ in age (*t*(40.6) = .98, *p* = .354), educational level (χ^2^ (3, *N =* 295) = 4.5, *p* = .212), level of functioning (*t*(45.4) = -.11, *p* = .913) or between different diagnoses (χ^2^ (6, *N =* 298) = 8.15, *p* = .227). The reasons for the interest in online self-management interventions are shown in Fig 5. All results regarding demographic aspects and diagnoses are presented in Table 2.

**Table 2:** Interest in and use of online self-management interventions.

	Interest in online self-management interventions			Use of online self-management interventions		
	Yes	no	*P*	yes	no	*p*
**Age** (yr) (Mean, *SD*)	33.75 (10.88)	39.28 (14.72)	.001*	35.27 (10.70)	37.26 (13.78)	.444
**GAF** (%) (Mean, *SD*)	58.57 (11.83)	57.15 (11.09)	.301	57.63 (7.95)	57.46 (12.01)	937
**Duration of internet use** (Mean, SD)	24.04 (19.36)	18.11 (14.90)	.006*	22.75 (20.40)	20.41 (16.88)	.497
**Gender**			.309			.001*
Female (*n*, %)	77 (48.7)	81 (51.3)		25 (15.1)	141 (84.6)	
Male (*n*, %)	52 (42.6)	70 (57.4)		5 (3.8)	127 (96.2)	
**Education level**			.003*			.212
Mandatory school (*n*, %)	39 (34.8)	73 (65.2)		11 (8.9)	112 (91.1)	
High school degree (*n*, %)	46 (52.3)	42 (47.7)		7 (7.6)	85 (92.4)	
University degree (*n*, %)	42 (59.2)	29 (40.8)		12 (16.2)	62 (83.8)	
**Diagnoses according to ICD-10**			.056			.267
**F10-F19** (disorders due to psychoactive substance use) (*n*, %)	6 (54.5)	5 (45.5)		1 (9.1)	10 (90.9)	
**F20-F29** (schizophrenia, schizotypal, and delusional disorders) (*n*, %)	12 (52.2)	11 (47.8)		0 (0)	25 (100)	
**F30-F39** (affective disorders) (*n*, %)	55 (37.2)	93 (62.8)		22 (14.1)	134 (85.9)	
**F40-F49** (neurotic, stress-related and somatoform disorders (*n*, %)	31 (51.7)	29 (48.3)		5 (7.8)	59 (92.2)	
**F50-F59** (behavioural disorders with physical disorders) (*n*, %)	3 (75)	1 (25)		0 (0)	4 (100)	
**F60-F69** (disorders of adult personality and behaviour) (*n*, %)	14 (63.6)	9 (36.4)		2 (8.7)	21 (91.3)	
**F90-F98** (behavioural and emotional disorders beginning in childhood and adolescence) (*n*, %)	8 (66.7)	4 (33.3)		0 (0)	15 (100)	
**Did patient find online services already used to be helpful?**			.033*			410
Yes (*n*, %)	105 (48.6)	111 (51.4)		23 (10.3)	201 (89.7)	
No (*n*, %)	11 (29.7)	26 (70.3)		6 (14.6)	35 (85.4)	

Statistically significant value is marked with * (p<0.05).

yr = years; SD = standard deviation; n = sample size

## Discussion

### Relevant findings

Our findings emphasize the role the internet plays for patients who are dealing with mental disorders: 88.8% of the participants had used the internet to inform themselves about mental health-related topics. This corresponds with the general utilization of the internet (98.3%) [29]. This number has increased within five years from 70.9% found in an earlier research with a similar sample in Germany [23]. Participants especially used generalist sites such as search engines (66.2%) and Wikipedia (21%) rather than websites of hospitals or professional societies (7.2%) to inform themselves about mental health-related topics. While only a few searched for contact with other patients (20.3%) or forums conducted by a professional (15.9%), patients mostly searched for general information about mental disorders (75.4%), medication (56.5%) and contact to psychiatrists and clinics (56.1%). This underlines the internet’s role as a source of information and a gatekeeper to professional mental health care, that is especially used by patients with a lower level of functioning. In our survey, 90.3% of the participants possessed a smartphone, for the advantages of mobile interventions, which compared to computer-based services offer a higher flexibility and availability, thus easing the approach for patients with mental disorders [30].

In total, only 10.1% of the sample had used online self-management interventions before even though we found the interest in online self-management interventions to be much greater (46.1%). Our findings show that patients would like to use online self-management for possibilities of prevention and self-monitoring (34.3%) and the availability of the service (34.3%). We did not find differences in the use of online self-management interventions and different diagnoses or any demographic aspects except gender, showing women to be currently using online self-management interventions more often than men.

### Link with others’ findings

The great potential of the internet as a link between patients and the health care system has been shown in projects such as the Australian MindSpot Clinic [31]. Our results correlate to several studies showing the capability of web-based services to lower barriers of getting involved into a professional treatment [32,33]. Coinciding with the findings of earlier studies, younger participants and patients on a lower level of functioning were more interested in finding information about their diagnoses or searching for exchange with other patients and professionals [34,35].

Though the general use of online services is quite common among patients with mental disorders, the knowledge about online self-management interventions is low, due to the confusing number of services available, but also the lack of accessibility of proven services [36]. While we did not find differences in the interest and utilization of internet interventions among patients with different diagnoses, current mental health services found online are focussing mostly on depression and anxiety, while other disorders are left behind (i.e. schizophrenia, substance abuse) [37]. Research regarding web-based services for patients suffering from schizophrenia also found positive outcomes in the treatment and emphasized the potential of the internet for these disorders [38–40].

Web-based services and interventions can not only provide effective help for the patients, but also for caregivers, relatives and informal caregivers [41]. These services highlight another aspect of E-mental health that is focussed on the support of caregivers and has both direct and indirect influence on the treatment of the patient [42].

Online self-management interventions are not only assigned to meet the patients’ interests, but also have to suit the work of professional therapists [34]. Furthermore, as internet-based interventions are being used more often, assessing the outcome of these interventions will become increasingly important [43].

### Implications

Web-based services offer new ways for patients to get informed about mental health-related topics adding the advantages of flexibility, availability and anonymity to the range of therapeutic attempts, especially for younger patients. This suggests a differing approach between age groups in dealing with their mental illness [44].

At the moment, treatment options developed and discussed in research are not fully implemented into the health care system: Our data reveals a gap between utilisation and demand, which might derive from various reasons e.g. the lack of standardized regulations for quality and data protection or the readiness of both the patient and the therapist to get involved in web-based therapeutic attempts [45]. According to our findings, the interest to use online self-management interventions is influenced by the age, gender and education level of the patient.

### Limitations

Firstly, the recruitment strategy raises the possibility of self-selection bias, self-referral, and motivation for time investment. Another limitation of our research is that participants were recruited from a single centre in Germany. Due to the fluctuation at the university hospital, it was not possible to include all patients at the same time into the study. It is noteworthy that patients from one infirmary were introduced to online self-management interventions (iFightDepression) during their treatment which might have influenced the evaluation of online self-management by those patients. Since the treatment and the execution of the study were not connected in any way, all questionnaires were evaluated equally. Certain methodological limitations need to be addressed. Firstly, the cross-sectional study design precludes any causal conclusions, thus limiting the generalizability of the findings. Furthermore, there is no standardized method for the measurement, thus the questionnaire used in our survey had to be modified to meet the objectives of the study.

## Conclusion

While the internet is an important part in the everyday life of patients with mental disorders, web-based treatment options do not yet exploit their full potential. Finding information about their illness is most important to patients and certain demographic and clinical parameters (e.g. age, gender or level of functioning) define key groups which might be addressed by online services in specific ways. Even though there is a great range of services and a lot of research on this matter, further steps must be taken to implement these services into the treatment of patients with mental disorders [46]. This way, online services could fulfil preventative functions, lower barriers to get professional help, and accompany professionals and patients during the treatment. In our sample online self-management interventions were used regardless of the patients’ diagnosis, suggesting that all patients suffering from mental illnesses could benefit from these interventions. Our results show a great interest to use online self-management interventions, yet the number of patients who use online self-management interventions is much lower. Reasons for this finding should be analysed in the future by evaluating how web-based services can be integrated into the patients’ treatment; e.g. by informing them about these services at different stages of their therapy and investigating their utilization. To improve the standard of such investigations we suggest the compilation of a standardized questionnaire.

## Supporting information

S1 File Questionnaire—Utilization of e mental-health and online self-management interventions. (DOCX)

S2 File Questionnaire (German)—Erfassung des internetnutzungsverhaltens. (DOCX)

S3 File Dataset and Syntax (SAV-format). (SAV)
