# Supplementary material for: Utilization of e-mental-health and online self-management interventions of patients with mental disorders—A cross-sectional analysis
Source: PLoS One. 2020 Apr 20;15(4):e0231373. doi: 10.1371/journal.pone.0231373 (PMC7170258; doi:10.1371/journal.pone.0231373)
Supplement: S1 File — (DOCX) [file pone.0231373.s001.docx]

Patientennummer: Datum:

Erfassung des Internetnutzungsverhaltens

bei Patienten mit psychischen Erkrankungen

erstellt von:

Carolin Webelhorst

carolin.webelhorst@medizin.uni-leipzig.de

Sehr geehrte Patientin, sehr geehrter Patient,

bitte füllen Sie den Fragebogen vollständig aus. Die von Ihnen gemachten Angaben werden anonymisiert ausgewertet.

Herzlichen Dank für Ihre Mitarbeit!

# Angaben zur Person

Geschlecht: m w

Alter:

Behandlung: stationär teilstationär ambulant

Diagnose: ______________________________________

Familienstand:

- ledig
- verheiratet oder mit Partner/In zusammenlebend
- geschieden/ getrennt
- verwitwet

Höchster Schulabschluss:

- noch in der Schule
- kein Schulabschluss
- Realschule/ mittlere Reife
- Abitur/ Fachabitur
- Abgeschlossenes Hochschulstudium

Aktuelle berufliche Situation:

- Auszubildende(r)
- Student/In
- Arbeitslose(r)
- Angestellte(r) oder Beamte(r)
- Selbstständige(r)
- Hausfrau/-mann
- Rentner/In
- Andere

# Fragen zum Internetgebrauch

## **Haben Sie das Internet schon einmal genutzt?**


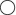
 Ja
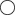
 Nein

## **Falls nein, warum nicht?**

_______________________________________________________________________

_______________________________________________________________________

**! Für Patienten, die das Internet noch nicht benutzt haben, weiter mit Frage 3 (Seite 7)!**

**2.3 Seit wie vielen Jahren nutzen Sie bereits das Internet?**  seit ______ Jahren

**2.4 Wie viele Stunden pro Woche haben Sie in den letzten zwölf Monaten durchschnittlich privat das Internet genutzt?**  __________ Stunden pro Woche

**2.5 Besitzen Sie ein Smartphone mit Internetzugang?**

Ja Nein

**2.6. Besitzen Sie ein Tablet mit Internetzugang?**

Ja Nein

**2.7 Welche Geräte verwenden Sie zur Nutzung des Internets? (Bitte ankreuzen und dahinter Angabe in Prozent, in Summe nicht mehr als 100%)**

Desktop-PC % Laptop %

Tablet % Smartphone %

**2.8 Welche Internetdienste nutzen Sie? (Bitte ankreuzen, mehrere Kreuze möglich)**

Chat

soziale Netzwerke, und zwar:

Facebook

sonstige Communities (z.B. VZ-Netzwerke, google+ etc.)

Blogs

Diskussionsforen

E-Mail

Downloads von Dateien (Musik, Filme etc.)

Suchmaschinen

Spiele

Einkaufen (z.B. Bücher, Kleidung)

Nachrichten

berufliche/schulische Informationen

Freizeitinformationen (z.B. über Reisen)

Online-Banking

Fernsehen, Radio hören

Partner-, Kontaktbörsen

Sonstiges:

**2.9 Haben Sie schon einmal Internetseiten mit medizinischen Inhalten besucht?**

Nein

Ja, und zwar zu folgenden Themen:

**2.10 Haben Sie sich schon einmal im Internet über psychische Erkrankungen informiert?**

Nein

Ja, und zwar zu folgenden Themen:

**2.11 Haben Sie sich schon einmal im Internet über Medikamente für psychische Erkrankungen informiert?**

Nein Ja

**2.12 Wenn ja, warum? (Mehrfachnennung möglich)**

O um mich vor dem Arztbesuch zu informieren

O um mich über Medikamente (z.B. Nebenwirkungen) zu informieren

O weil ich dem Arzt nicht vertraue

O weil ich den Arzt nicht verstanden habe

O weil mir die Informationen, die mir der Arzt gegeben hat, nicht ausreichten

O um meine Erfahrungen mit anderen Betroffenen auszutauschen (E-Mail, Chat)

O um einen Arzt zu finden

O um mit einem Arzt zu kommunizieren

O aus anderen Gründen, und zwar __________________________________________________

**2.13 Fanden Sie die Internetseiten zu psychischen Erkrankungen verständlich?**

Nein Ja

**2.14 Fanden Sie die Internetseiten zu psychischen Erkrankungen hilfreich?**

Nein Ja

**2.15 Hat Ihnen das Internet bei der Bewältigung Ihrer psychischen Erkrankung geholfen?**

Ja, weil

Nein, weil

Vielleicht, weil

**2.16 Haben Sie schon einmal psychiatrische Medikamente nicht genommen aufgrund einer voran gegangenen Internetrecherche?**

Nein Ja

**2.17 Haben Sie schon einmal psychiatrische Medikamente genommen aufgrund einer voran gegangenen Internetrecherche?**

Nein Ja

**2.18 Für welche psychiatrischen Themen und Inhalte interessieren Sie sich im Internet besonders? (Mehrfachnennung möglich)**

O Informationen zu bestimmten psychiatrischen Krankheitsbildern

O Informationen über Medikamente (z.B. Antidepressiva, Antipsychotika)

O Suche nach Psychiatern bzw. Psychiatrischen Kliniken (z.B. zur Kontaktaufnahme oder Terminvereinbarung)

O Erfahrungsaustausch mit anderen Betroffenen

O Foren, in denen Psychiater/Psychotherapeuten Fragen von Betroffenen beantworten, o.ä.

O sonstiges, und zwar

**2.19 Welche Internetseiten besuchen Sie zum Thema psychische Erkrankungen? (Auch die Angabe von Stichwörtern für Suchmaschinen ist möglich.)**

www.

www.

www.

**2.20 Sind Sie der Meinung, dass es mehr Internetseiten geben sollte, die sich mit psychischen Erkrankungen beschäftigen?**

Nein Ja

**2.21 Haben Sie schon einmal über das Internet mit einem Psychiater/Psychotherapeuten Kontakt aufgenommen?**

Nein Ja

**2.22 Wenn nein, glauben Sie, dass das Internet Ihnen diese Kontaktaufnahme erleichtern würde?**

Ja Nein Weiß nicht

**2.23 Wünschen Sie sich Internetangebote zur Unterstützung im Umgang mit Ihrer psychiatrischen Erkrankung (sog. Selbstmanagement)?**

Nein Ja, weil:

**2.24** **Haben Sie schon einmal ein Internetangebot oder eine Selbstmanagement- App (z.B. iFightDepression, Deprexis) genutzt?**

Nein Ja, welche
