# Supplementary material for: Utilization of e-mental-health and online self-management interventions of patients with mental disorders—A cross-sectional analysis
Source: PLoS One. 2020 Apr 20;15(4):e0231373. doi: 10.1371/journal.pone.0231373 (PMC7170258; doi:10.1371/journal.pone.0231373)
Supplement: S2 File — (DOCX) [file pone.0231373.s002.docx]

Patient No: Date:

Utilization of e mental-health and online self-management interventions

- Questionnaire -

conducted by:

Carolin Webelhorst

carolin.webelhorst@medizin.uni-leipzig.de

Dear participant,

please complete the questionnaire on your own. Your answers are anonymized throughout the whole process of analysis.

Thank you for your participation!

# Personal information

Gender: m f

Age:

Treatment: inpatient day hospital outpatient

Diagnosis: ______________________________________

Marital status:

- unwed
- married or in a partnership
- divorced or living separately
- widowed

Education level:

- still in school
- no degree
- Mandatory school degree
- High school degree
- University degree

Occupation:

- apprentice
- student
- unemployed
- employee or official
- self-employed
- housewife
- retiree
- other (volunteer, intern):

# Questions about your internet use

## **Have you ever used the internet?**


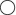
 Yes
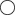
 No

## **If not, why?**

_______________________________________________________________________

**! Patients who have never been using the internet, continue with question 2.24 (page 6)!**

**2.3 Since how many years have you been using the internet?**  since______ years

**2.4 How many hours per week did you spend on the internet for private matters in the last month?**  __________ hours per week

**2.5 Do you own an internet-enabled smartphone?**

yes no

**2.6. Do you own an internet-enabled tablet?**

yes no

**2.7 Which devices do you use to access the internet? (Percentual use, in total not more than 100%)**

Desktop-PC Laptop Tablet Smartphone

**2.8 What do you use the internet for? (check boxes, multiple answers possible)**

Chat

Social media:

Facebook

other communities (e.g. Instagram, google+ etc.)

Blogs

forums

E-Mail

Downloading/ Streaming (music, films etc.)

Search engines

Games

Shopping (e.g. books, clothing)

News

Professional/ educational Information

Information about free time activities (e.g. travelling)

Online-Banking

Online-TV, Online-Radio

Online dating

other:

**2.9 Have you ever visited websites with medical content?**

No

Yes, I’ve been searching on these topics:

**2.10 Have you ever been searching for information about mental illnesses on the internet?**

No

Yes, I’ve been searching on these topics:

**2.11 Have you ever been searching for information about medication for mental illnesses on the internet?**

No Yes

**2.12 If yes, why? (multiple answers possible)**

O do get informed before visiting a doctor

O to get informed about medication (side effects)

O because I do not trust the doctor

O because I did not understand the doctor

O because the information provided by the doctor was not sufficient

O to exchange my experiences with others (E-Mail, Chat)

O to find a doctor

O to communicate with my doctor

O other reasons: _________________________________________________

**2.13 Were the websites on mental illnesses comprehensible in your opinion?**

No Yes

**2.14 Were the websites on mental illnesses helpful in your opinion?**

No Yes

**2.15 Did the internet help you to cope with your mental illness?**

Yes, because

No, because

Maybe, because

**2.16 Have you ever not taken psychiatric medication due to a prior internet research?**

No Yes

**2.17 Have you ever taken psychiatric medicine because of a prior internet research?**

No Yes

**2.18 In which psychiatric topics are you interested on the internet? (multiple answers possible)**

O Specific information about certain disorders

O Information about medication (e.g. anti-depressives, antipsychotics)

O Search for professionals, psychiatrists or psychiatric hospitals (e.g. to make contact/ appointment)

O Exchange of experiences with others

O forums conducted by a professional (e.g. psychiatrist)

O others

**2.19 Which websites about mental illness did you visit? (including keywords for search engines)**

www.

www.

www.

**2.20 Do you think, there should be more websites that are focussed on mental illnesses?**

No Yes

**2.21 Have you ever contacted a psychiatrist or psychotherapist via the internet?**

No Yes

**2.22 If not, do you think it would be easier to make contact via the internet?**

Yes No I don’t know.

**2.23 Would you like more online services to cope with your mental illness (so called online self-management interventions)?**

No Yes, because:

**2.24** **Have you ever used online self-management interventions or (e.g. iFightDepression, Deprexis)?**

No Yes, I have used:
